# A Novel Solution for the High Defibrillation Threshold in Patients with a DF-4 Lead: Adding a High Voltage Adaptor/Splitter

**DOI:** 10.1016/s0972-6292(16)30756-2

**Published:** 2014-05-25

**Authors:** Shmuel Inbar, Srikanth Seethala

**Affiliations:** Department of Internal Medicine, University of New Mexico School of Medicine, Albuquerque, NM

**Keywords:** DF4 adaptor/splitter, high DFT, subcutaneous shocking lead

## Abstract

A high defibrillation threshold occurs in approximately 6% of implants. The defibrillation threshold can be improved by addition of a defibrillation lead. However, the DF-4 high energy ICD header precludes the addition of a defibrillation lead. Here we report on use of a new high voltage adaptor/splitter that enables the addition of an extra defibrillation lead.

## Introduction

In March 2010, the International Organization for Standardization (ISO) formalized a new four-pole, in-line connecting system standard, the DF-4 high energy lead. [1,2] An Implantable Cardioverter Defibrillator (ICD) header that complies with the new standard has one port that accommodates the coils and the ventricular pace/sense connectors. This DF-4 system provides the advantages of easy connection and removal of the lead and smaller header size, while avoiding a mismatch of the high voltage connectors. A limitation of this new standard, however, has been the inability to add ventricular pacing or defibrillation leads to the header. This report describes the use of a new, novel adaptor/splitter that enables connecting a defibrillating lead without the need to replace the ICD generator.

## Case Report

A 47-year-old male with a history of ischemic cardiomyopathy received an ICD for spontaneous, hemodynamically unstable sustained Ventricular Flutter (VF). The ICD was a Medtronic Protecta VR with a quadripolar DF-4 single coil lead (model 6935M). The maximum shock energy output for this generator is 35 Joules (J). Intraoperative induced ventricular tachycardia (VT) at cycle length (CL) of 300 msec was terminated by Anti Tachycardia Pacing (ATP). A subsequent induced VF was terminated by a 15 J shock on the first attempt.

**Figure 1:**
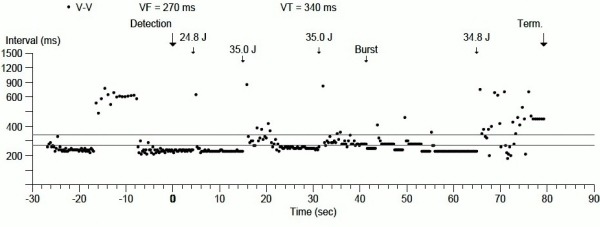
Arrhythmias and ICD therapies displayed as cycle length over time.

**Figure 2:**
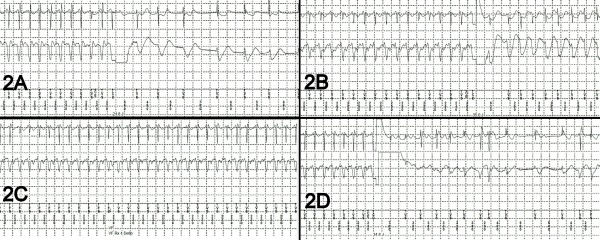
Electrocardiograms of arrhythmias and response to therapy. See text.

A month later, while exercising and after skipping few doses of his heart failure medications, the patient sustained five episodes of shocks (Figure 1). Arrhythmia interrogation revealed sustained VT at a CL of 230-240 msec, which was terminated with a 24.8 J shock (Figure 2A). However, immediately after the initial episode, VT had recurred at a CL similar to first episode. A 24.8 J shock was delivered which resulted in a stable VT at a CL of 230 msec and what appeared to be a slightly different morphology. A 35 J shock was delivered (Figure 2B), which resulted in a stable VT at a CL of 250 msec that was morphologically different from previous episodes. Another shock at 35 J was then delivered, with another change in the VT morphology and stabilization of cycle length at 280 msec, which was in the VT therapy zone. In an attempt to terminate the tachycardia, ATP was deployed 3 times resulting in acceleration of the VT to a CL of 230 msec (Figure 2C). Another shock was delivered at 35 J, which resulted in successful termination of VT after 8 beats with variable CL (Figure 2D). Bursts of polymorphic VT were noted after termination of the VT. Thus, 2 shocks at maximum output did not terminate the VT, and only the third shock at 35 J resulted in an "unclean" termination of the arrhythmia. Mexiletine 200 mg twice daily was started and patient was referred to our institution for further management.

After careful interrogation, and failure to identify any reversible causes of the increase in defibrillation thresholds (complete metabolic profile, cardiac enzymes), Mexiletine was discontinued due to concerns that it could further increase the defibrillation threshold. [3] It was felt that there was not a sufficient margin of safety in the defibrillation threshold, and implantation of subcutaneous shocking lead was planned.

**Figure 3:**
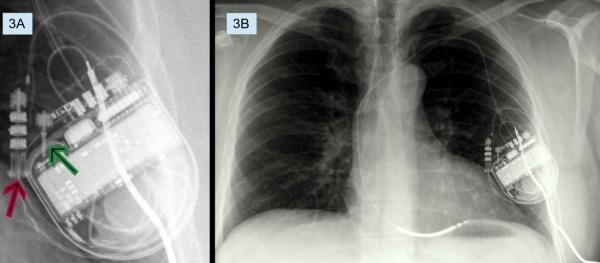
A: DF-4 adaptor/splitter. Red arrow points to the DF-4 port of the adaptor/splitter to which the RV defibrillation lead is connected. Green arrow points to the DF-1 port of the adaptor/splitter to which the subcutaneous defibrillation electrode is connected. B: PA view showing subcutaneous defibrillation electrode connected to the adaptor/splitter.

Since adding another lead is not a possibility with the DF-4 header, we arrange with a manufacturer to get a pre-production adaptor/splitter (Figure 3A) with bifurcated DF-1 and DF-4 inputs into the ICD's DF-4 header. A single-coil DF-1 subcutaneous defibrillation electrode (Medtronic 6996SQ) was tunneled from the left precordial area to the left axilla (Figure 3B). The new DF-1 lead and the existing DF-4 lead were both connected to the bifurcated adaptor, which, in turn, was connected to the DF-4 port of the original ICD generator.

Intraoperative VF was successfully induced with shock on T. The first shock, at 15 J, successfully terminated the VF and sinus rhythm emerged. The measured shocking impedance was 53 ohms. A second arrhythmia induction resulted in VT at a CL of 280 msec. Five attempts of ATP were deployed with eventual degeneration of the VT into ventricular fibrillation. A 7.9 J shock failed to defibrillate, but a 15 J shock successfully terminated the ventricular fibrillation. A third induction resulted in VT at cycle length of 290 msec, which was terminated with an 8 J shock. ATP was removed from the therapies because of its ineffectiveness and pro-arrhythmic effects. Repeat interrogation a month later revealed no changes in the defibrillation lead impedance, pacing lead impedance, or pacing thresholds.

## Discussion

The DF-1 standard was designed in early 1990s in order to establish industrial standards and uniformity in lead-generator interface. [2] Depending on the number of defibrillating coils, the ICD leads are either bifurcated or trifurcated at the proximal end that fits into the generator header. The major drawbacks of this DF-1 lead system are the bulky ICD header and lead yoke, and a concern of possible error in connecting the high voltage pins to the wrong ports in the header. In addition, the multiple set screws of the DF-1 connector create potential for problems. The DF-4 standard addresses these shortcomings. In addition, the DF-4 header offers more reliable long-term sealing by locating sealing rings within the header rather than in the lead, as was the case with the DF-1 standard. Thus, new seals are introduced with the replacement of every DF-4 generator.

However, the DF-1 header offered several advantages over the new DF-4 standard. The DF-1 header permitted the addition an extra sense/pace lead using a Y adaptor. The DF-1 standard also enabled an ICD to be downgraded to a pacemaker easily. Such downgrading may be advisable in pacing-dependent patients, or CRT responders that do not wish to have ICD therapies anymore. In addition, the DF-1 header allowed the proximal coil pin to be replaced with a stand-alone defibrillation lead. The design of the DF-4 header has so far not offered a solution that enables the addition of a defibrillation lead.

At times it may be important to be able to add a defibrillation lead in order to improve defibrillation thresholds. High defibrillation thresholds (HDT), defined as <10 J safety margin from maximum output, are not uncommon; they are reported in two large studies to be around 6% of ICD implantations. [4,5]

HDT etiology can be divided into patient-related, device-related, metabolic, medication, and procedure-related. Pre-implantation predictors for HDT are amiodarone therapy, male gender, obesity, left ventricular hypertrophy, and a wide QRS complex. [6-9] Procedure- related factors include loose connections, pocket hematoma, device location, and ICD lead micro-dislodgement. Device-related factors include shocking waveform morphology, duration, polarity, and defibrillation vectors. [7]

Some strategies have been developed to reduce HDT. In some defibrillators, shocking waveform tilt can be modified, which may reduce the defibrillation threshold. [10] Changing the defibrillation vector can result in lowering the defibrillation threshold. The defibrillation vector can be changed by repositioning the implanted lead, or adding another defibrillation lead. Some of the options for placement of an added lead are in the azygous vein, in the coronary sinus, or subcutaneously. The choice of such lead placement depends on the patient's anatomy and the operator's experience.

With the DF-1 header technology, an extra defibrillation lead could be connected to the header by a replacing the proximal coil connector in the header, or by connecting both the proximal coil and the extra lead with a Y connector.10 The single port design of the new DF-4 technology eliminates the possibility of adding an extra coil or lead. In nearly 0.14% of ICD implants, a DF-4 generator has been replaced by an DF-1 system due to an inadequate defibrillation safety margin and inability to improve it by adding a lead. [11,12] The high voltage splitter/adaptor was designed to address this specific issue. Here we report a successful implantation of a subcutaneous defibrillation lead by using an adaptor/splitter to connect to a DF-4 ICD header. This adaptor/splitter will solve one of the major drawbacks of this new technology by enabling the addition of a defibrillation lead. However, adaptors have not yet been developed to address connectivity of a pace/sense lead to a DF-4 system or to use an existing DF-4 lead during downgrading of an ICD to a pacing-only system.

## References

[R1] Haqqani HM (2009). The implantable cardioverter-defibrillator lead: principles, progress, and promises. Pacing Clin Electrophysiol.

[R2] Sticherling C (2012). Introduction of new industry standards for cardiac implantable electronic devices: balancing benefits and unexpected risks. Europace.

[R3] Dopp AL (2008). Effect of drugs on defibrillation capacity. Drugs.

[R4] Russo AM (2005). Defibrillation threshold testing: is it really necessary at the time of implantable cardioverter-defibrillator insertion?. Heart Rhythm.

[R5] Osswald BR (2009). High defibrillation threshold in patients with implantable defibrillator: how effective is the subcutaneous finger lead?. Eur J Cardiothorac Surg.

[R6] Kashani A (2005). Significance of QRS complex duration in patients with heart failure. J Am Coll Cardiol.

[R7] Rahaby M (2012). Bailout Strategy for High Defibrillation Thresholds. The Journal of Innovations in Cardiac Rhythm Management.

[R8] Jacob S (2010). High defibrillation threshold: the science, signs and solutions. Indian Pacing Electrophysiol J.

[R9] Verma A (2010). : Incidence of very high defibrillation thresholds (DFT) and efficacy of subcutaneous (SQ) array insertion during implantable cardioverter defibrillator (ICD) implantation. J Interv Card Electrophysiol.

[R10] Mainigi SK (2006). How to manage the patient with a high defibrillation threshold. Heart Rhythm.

[R11] Cantillon DJ (2013). Clinical Experience and Procedural Outcomes Associated with the DF4 Implantable Cardioverter Defibrillator System: The SJ4 Postapproval Study. Pacing Clin Electrophysiol.

[R12] Cogert GA (2012). Limitations of the DF-4 defibrillator connector necessitating device removal. Pacing Clin Electrophysiol.

